# Assessment of the Performance of Magnetic Resonance Imaging/Ultrasound Fusion Guided Prostate Biopsy against a Combined Targeted Plus Systematic Biopsy Approach Using 24-Core Transperineal Template Saturation Mapping Prostate Biopsy

**DOI:** 10.1155/2016/3794738

**Published:** 2016-05-16

**Authors:** Francis Ting, Pim J. Van Leeuwen, James Thompson, Ron Shnier, Daniel Moses, Warick Delprado, Phillip D. Stricker

**Affiliations:** ^1^St. Vincent's Prostate Cancer Centre, Darlinghurst, NSW 2010, Australia; ^2^Garvan Institute of Medical Research, The Kinghorn Cancer Centre, Darlinghurst, NSW 2010, Australia; ^3^School of Medicine, University of New South Wales, Kensington, NSW 2052, Australia; ^4^Southern Radiology, Randwick, NSW 2031, Australia; ^5^Spectrum Radiology, Randwick, NSW 2031, Australia; ^6^Douglas Hanly Moir Pathology, Macquarie Park, NSW 2113, Australia

## Abstract

*Objective.* To compare the performance of multiparametric resonance imaging/ultrasound fusion targeted biopsy (MRI/US-TBx) to a combined biopsy strategy (MRI/US-TBx plus 24-core transperineal template saturation mapping biopsy (TTMB)).* Methods.* Between May 2012 and October 2015, all patients undergoing MRI/US-TBx at our institution were included for analysis. Patients underwent MRI/US-TBx of suspicious lesions detected on multiparametric MRI +/− simultaneous TTMB. Subgroup analysis was performed on patients undergoing simultaneous MRI/US-TBx + TTMB. Primary outcome was PCa detection. Significant PCa was defined as ≥Gleason score (GS) 3 + 4 = 7 PCa. McNemar's test was used to compare detection rates between MRI/US-TBx and the combined biopsy strategy.* Results.* 148 patients underwent MRI/US-TBx and 80 patients underwent MRI/US-TBx + TTMB. In the MRI/US-TBx versus combined biopsy strategy subgroup analysis (*n* = 80), there were 55 PCa and 38 significant PCa. The detection rate for the combined biopsy strategy versus MRI/US-TBx for significant PCa was 49% versus 40% (*p* = 0.02) and for insignificant PCa was 20% versus 10% (*p* = 0.04), respectively. Eleven cases (14%) of significant PCa were detected exclusively on MRI/US-TBx and 7 cases (8.7%) of significant PCa were detected exclusively on TTMB.* Conclusions.* A combined biopsy approach (MRI/US-TBx + TTMB) detects more significant PCa than MRI/US-TBx alone; however, it will double the detection rate of insignificant PCa.

## 1. Introduction

The generally accepted diagnostic approach for prostate cancer (PCa) involves performing random biopsies based on abnormal digital rectal examination (DRE) and prostate specific antigen (PSA) levels. However, this approach is limited by the fact that a negative DRE does not necessarily exclude PCa [1, 2] and although the risk of high-grade PCa increases with PSA level, there is no serum PSA threshold that can completely exclude significant PCa [3]. The drawbacks of the random systematic, template-based sampling approach are the potential for missing significant PCa and over-sampling of insignificant PCa [4]. Furthermore, random biopsies underestimate tumour grade compared with radical prostatectomy (RP) 30–40% of the time [5, 6].

Targeted biopsy to suspicious lesions on multiparametric magnetic resonance imaging (mp-MRI) has emerged with the potential advantages of increased diagnostic accuracy and fewer cores. It allows the sensitivity and negative predictive value (NPV) of mp-MRI to be utilised with the real-time capabilities of transrectal ultrasound. There are a number of MRI-targeted biopsy methods, which include in-gantry/in-bore MRI guided biopsy, MRI/ultrasound fusion targeted biopsy (MRI/US-TBx), and cognitive guided biopsy. Much of the rapidly growing body of literature on MRI-targeted biopsy has compared MRI/US-TBx to 10–12 core transrectal ultrasound guided biopsy (TRUSGB) and systematic reviews and meta-analysis have concluded that MRI/US-TBx detects more significant PCa [7, 8] and less insignificant PCa [9] compared with TRUSGB.

In order to improve standardisation of the reporting and interpretation of this continually growing body of literature, the standards of reporting for MRI-targeted biopsy studies (START) of the prostate were published in 2013 [10], written by an international working group. In addition, the European Society of Urogenital Radiology (ESUR) published the PI-RADS scoring system [11] in order to standardise the interpretation and reporting of prostate mp-MRI, and subsequently this scoring system has been validated in both primary and repeat biopsy cohorts [12–15].

Recent studies have utilised transperineal template saturation mapping biopsy (TTMB) as a reference test [16–18] in evaluation of MRI/US-TBx given the limited sensitivity and concordance with prostatectomy specimens of standard 10–12 core TRUSGB [19]. The consensus has been that the gold standard for cancer detection in primary biopsy is a combination of systematic and targeted cores. Our study aims to build on the current body of evidence by comparing the performance of MRI/US-TBx to that of a combined biopsy strategy (TTMB + MRI/US-TBx) in a meaningful way by adhering to START and PI-RADS guidelines.

## 2. Methods

### 2.1. Patient Selection and Study Design

Between May 2012 and October 2015, all patients undergoing MRI/US-TBx aged >40 years, with abnormal PSA or DRE, were included for retrospective study analysis. Patients underwent mp-MRI to identify regions suspicious for PCa followed by MRI/US-TBx of suspicious lesions ± simultaneous 24-core TTMB. Indications for MRI/US-TBx included previous negative biopsy, region of interest (ROI) on mp-MRI <1.5 cm, or ROI located in an unusual location not included in a standard TTMB template. A subgroup analysis was performed on all patients undergoing simultaneous MRI/US-TBx and TTMB. Informed consent was obtained from all patients and data collection performed as part of an approval from our institutional Human Research Ethics Committee (SVH File Number 12/231).

### 2.2. Imaging Protocol

All mp-MRIs were performed at 2 centres. Centre 1 used a 1.5 Tesla magnet (*b*-value 0–800 s/mm^2^) and Centre 2 used a 3 Tesla magnet (*b*-value 0–1500 s/mm^2^). Mp-MRI was performed using a standard MRI protocol described in [13]. Previous analysis has shown that there is no significant difference in diagnostic performance between these two centres [13]. All mp-MRIs were supervised and reported by subspecialised uroradiologists with at least 12 years of experience in >3000 cases each (RS and DM) following the standardised 5-point Prostate Imaging Reporting and Data System (PI-RADS) scale. Regions of interest (ROIs) were assigned a score of 1 to 5 for each parameter (T2WI, DCEI, and DWI) and then based on these individual parameter scores an overall impression ROI score was given. MRI-derived ROIs were assigned to twenty-seven regions of each prostate according to the standardised prostate reporting scheme [20]. ROI size was measured as the maximal diameter as taken from axial slices on the mp-MRI.

### 2.3. Biopsy Methodology

TTMB and MRI/US-TBx were conducted by a single urologist (PS) who had full access to all mp-MRI data and was aware of the location of ROIs for both systematic and targeted biopsies. MRI/US-TBx was generally conducted first followed by TTMB.

#### 2.3.1. TTMB

Transperineal, grid-directed template mapping biopsy was used as the reference test and was taken from 18 template locations using a modified Barzell technique [21]. A median of 24 cores were taken per patient and adjusted for volume with relative periurethral zone sparing.

#### 2.3.2. MRI/US-TBx

MRI/transrectal ultrasound (TRUS) fusion biopsy was performed with a floor mounted, transperineal grid TRUS platform (BK Medical, Herlev, Denmark) combined with Biojet rigid MRI/TRUS fusion software (Meditron, Melbourne, Victoria, Australia). The biopsy operator had access to all mp-MRI data with radiologist marked ROIs in digital format. These ROIs were sampled under live MRI/TRUS fusion visualisation with at least 2 cores taken, adjusted for ROI size.

Targeted and systematic cores were labelled and reported separately by a single experienced uropathologist (WD).

### 2.4. Statistical Methods

Primary outcome was PCa detection (overall, significant, and insignificant). Significant PCa was defined as ≥Gleason score (GS) 3 + 4 = 7 PCa. Gleason scores were compared between MRI/US-TBx and TTMB. GS upgrading on MRI/US-TBx compared to TTMB was defined as significant when the change was from nil/insignificant PCa to significant PCa or from GS 3 + 4 = 7 to ≥GS 4 + 3 = 7.

McNemar's test was used to evaluate differences in detection rates of PCa between MRI/US-TBx, TTMB, and the combined biopsy strategy. Binary logistic multivariate regression analysis was used to assess the significance of ROI size in detection of significant PCa. Sensitivity, specificity, negative predictive value (NPV), and positive predictive value (PPV) of PI-RADS scoring system were calculated based on PI-RADS 1/2 being negative and PI-RADS 3–5 being positive, and the combined biopsy strategy was used as the gold standard. Statistical analyses were performed using SPSS® V21. A *p* value <0.05 was considered statistically significant.

## 3. Results

### 3.1. MRI/US-TBx Patient Cohort

Demographic and baseline characteristics of the 148 included patients can be found in Table 1. Of these 148 patients, 93 (63%) were undergoing primary biopsy and 55 (37%) were undergoing repeat biopsy with 24 (16%) patients having a previous positive biopsy. 185 ROIs were targeted in these 148 men. There were 28 (15%) lesions scored as PI-RADS 1 or 2, 80 (43%) as PI-RADS 3, 55 (30%) as PI-RADS 4 and 22 (12%) as PI-RADS 5 (Table 2). An increasing PI-RADS score correlated with an increasing PCa detection rate with the detection rate of significant PCa ranging from 7% for PI-RADS 1/2 ROIs up to 82% for PI-RADS 5 ROIs. Overall, there were 92 cancers detected with 22 (24%) of these being GS 3 + 3 = 6 and 70 (76%) of these being GS 3 + 4 = 7 or greater.

ROI size data is displayed in Table 3. For ROIs <10 mm, the detection rate of significant PCa on MRI/US-TBx was 29% versus 56% for ROIs 10 mm or greater. Overall detection rate of PCa on MRI/US-TBx was 43% versus 62% for ROIs <10 mm versus ROIs 10 mm or greater, respectively. On multivariate analysis including age, PSA, DRE, primary versus repeat biopsy, prostate volume and PI-RADS score, ROI size was not significant (*p* = 0.09) but increasing age (*p* = 0.001) and PI-RADS score ≥4 (*p* = 0.02) were predictive in detection of significant PCa.

### 3.2. Comparison of Cancer Detection Performance: MRI/US-TBx versus Combined Biopsy Strategy

Eighty patients underwent both MRI-TBx and TTMB simultaneously. In this subgroup analysis, 65 (81%) patients underwent primary biopsy and 15 (19%) underwent repeat biopsy. Baseline characteristics of this subgroup can be found in Table 1.

There were 55 (69%) patients diagnosed with PCa and 38 (45%) of these were significant PCa. The detection rate of significant PCa for MRI/US-TBx and combined biopsy strategy was 40% versus 49% (*p* = 0.02) and the detection rate of insignificant PCa for MRI/US-TBx and combined biopsy strategy was 10% versus 20% (*p* = 0.04), respectively (Table 4). The proportion of detected cancers being significant for each biopsy technique was 32/40 (80%) for MRI/US-TBx and 39/55 (71%) for combined biopsy strategy (Table 4). Gleason score distribution for different biopsy techniques is displayed in Table 5.

Sixteen (20%) patients had a GS upgrade on combined biopsy strategy compared to their MRI/US-TBx with seven (9%) being significant upgrades. These missed cases are described in Table 6. All cancers were Gleason 3 + 4 = 7 PCa with a maximum of 15% Gleason 4. In addition, the location of the significant cancers detected on TTMB but missed on MRI/US-TBx did not correlate to the location of the ROI on mp-MRI in five of these seven cases.

Sensitivity and negative predictive value (NPV) of PI-RADS (when considering PI-RADS 1-2 negative, PI-RADS 3–5 positive) for detection of significant PCa were 98% and 92%, respectively (Table 7). The PPV of PI-RADS for detection of significant PCa when considering PI-RADS ≥3, ≥4, and 5 as positive was 56%, 74%, and 100%, respectively.

### 3.3. Comparison of Cancer Detection Performance: MRI/US-TBx versus TTMB

Median number of cores taken per patient was 24 (IQR 21–29) for TTMB and 4 (IQR 3-4) for MRI/US-TBx. Sampling efficiency was in favour of MRI/US-TBx with 20% of MRI/US-TBx versus 8% of TTMB cores detecting significant PCa.

The detection rate of significant PCa for MRI/US-TBx and TTMB was 40% versus 35% (*p* = 0.48) and the detection rate of insignificant PCa for MRI/US-TBx and TTMB was 10% versus 25% (*p* = 0.01).

Twenty patients (25%) had a GS upgrade on MRI/US-TBx compared to their TTMB with 15 (19%) being significant upgrades. Eleven cases of significant PCa were detected exclusively on MRI/US-TBx and 7 cases of significant PCa (all Gleason 3 + 4 = 7 with ≤15% Gleason 4) were detected exclusively on TTMB.

## 4. Discussion

Our results show that a biopsy strategy combining TTMB with MRI/US-TBx can increase the detection rate of significant PCa when compared to MRI/US-TBx alone; however this combined biopsy strategy also doubled the detection of insignificant PCa when compared to MRI/US-TBx alone. This is in line with recent data from a direct prospective comparison between MRI/US-TBx and TTMB which has shown that MRI/US-TBx can detect as many GS 7 or greater tumours while simultaneously avoiding the detection of 44% of lower grade disease [17]. Another series (*n* = 50) comparing both cognitive and software based MRI registration to TTMB has shown slightly lower rates of significant disease detection (68% for software based fusion versus 76% for TTMB) [18], and similarly, in another series (*n* = 437) more significant PCa was found exclusively in TTMB than exclusively in MRI/US-TBx (52 versus 18 cases) [16]. Thus, if the aim is purely to maximise the detection of significant PCa the combination of TTMB with MRI/US-TBx still appears to be the most effective biopsy strategy at present. However, in the current series it should be noted that all additional significant cancers detected by the addition of TTMB to the MRI/US-TBx biopsy strategy were GS 3 + 4 = 7 with a maximum GS 4 component of 15%. Given the slow natural history and high 10-year PCa specific survival of GS 3 + 4 PCa [22], a delayed diagnosis in the course of careful follow-up is unlikely to have resulted in missing the window of curability.

TRUSGB can miss 20–30% of clinically significant cancers due to undersampling, particularly in the anteroapical region of the prostate [23]. MRI/US-TBx overcomes this deficiency by sampling the suspicious region and thus is likely to be more effective than TRUSB in the detection of significant PCa. Accordingly, observational trials have shown that MRI/US-TBx has a higher detection rate for clinically significant cancer [24–26], a higher detection rate for high-risk PCa [27], and a lower sensitivity for clinically insignificant cancer compared to TRUSGB (0.51 versus 0.81, resp.) [9]. In addition, MRI/US-TBx has been shown to be able to detect as many GS ≥7 PCa compared to TTMB [17]. In the current series, we demonstrated a detection rate of MRI/US-TBx for significant PCa of 40%. A number of significant cancers were missed by MRI/US-TBx but the majority of these were outside of the ROI targeted on mp-MRI, which indicates that MRI/US-TBx is still a procedurally valid technique in our centre.

### 4.1. Magnetic Resonance Imaging Targeted Biopsy Can Reduce PCa Overdiagnosis

The disadvantage of using TTMB alone is the increased detection of insignificant PCa, with rates of insignificant PCa detection for TTMB of up to 25% being reported [28–30], which is consistent with the 25% rate of insignificant PCa detection by TTMB in the current series. Compared to this, MRI/US-TBx alone in the current series had a 10% detection rate of insignificant PCa and thus a future biopsy approach that replaces TTMB with MRI/US-TBx will significantly reduce the overdiagnosis of insignificant PCa.

### 4.2. Validation of the PI-RADS Scoring System

A PI-RADS score of 1/2 appeared to be effective in ruling out most cases of significant PCa and a PI-RADS score of 4/5 was able to predict prostate cancer; the data we present further validates the current data on the predictive information that is provided by PI-RADS scoring in prostate mp-MRI [14, 15]. In patients with PI-RADS 3 “equivocal” ROIs there were a significant proportion (21%) of patients with significant PCa, and comparable rates of significant PCa (26–29%) [13, 14] in PI-RADS 3 ROIs have been reported which supports the rationale of performing MRI-TBx on all patients with PI-RADS 3–5 lesions on mp-MRI.

Centres of excellence have reported sensitivity of 86–96% [13, 14, 31–34], and NPV of 90–92% [14, 33, 35] for mp-MRI in detection of significant PCa. Our reported sensitivity and NPV of 98% and 92%, respectively, is similar to these results and suggests the possibility of avoiding random biopsy in patients with negative mp-MRI, provided that standards are established for accreditation, training, and reporting of mp-MRI. Lower sensitivities of 58–76% [36, 37] and NPV of 79–89% [36, 37] have been reported in smaller series and this interstudy variability can be part-attributed to the significant learning curve associated with interpretation of prostate mp-MRI of about 100 cases [38, 39]. However, interstudy variability of NPV is also influenced by the overall prevalence of cancer and needs to be taken into account when comparing studies.

### 4.3. Limitations

Firstly, the primary operator was not blinded to the ROI when performing random biopsies and therefore an accurate direct comparison between TTMB and MRI/US-TBx alone is not possible from this study given this confounding factor. However, we are still able to draw an accurate comparison between MRI/US-TBx versus the combined biopsy strategy, which gives this study clinical utility. Secondly, this study was retrospective in nature but as noted above we have strict, standardised mp-MRI and biopsy protocols in place. Thirdly, longer follow-up is required, particularly in the biopsy negative patients and our operator and radiologists were not blinded to radiological or clinical data as per routine clinical practice. Fourthly, there are inherent limitations in using biopsy as the reference test: MRI/US-TBx could have missed MRI targets despite software registration [40] and there is a percentage of upgrading that occurs on radical prostatectomy. However in this cohort using radical prostatectomy as the reference test was not feasible due to ethical issues and positive selection bias. Finally, the binary logistic regression analysis performed to assess significance of ROI size in detection of significant PCa was performed on a per-lesion rather than a per-patient basis. Some patients had multiple ROIs/lesions and the ensuing correlation effect was not accounted for with a mixed model analysis, which resulted in the *p* values being more significant than they should be. However, this does not change the interpretation of nonsignificance for ROI size in detection of significant PCa.

### 4.4. Strengths

One of the main strengths of this study was the use of TTMB as a reference test. TTMB was used as it has been demonstrated as a robust reference test [13, 41], localises the index lesion [42] in most men with clinically significant disease [25], and detects more clinically significant cancer than 12-core TRUSGB [28]. Furthermore, another series demonstrated that in a significant proportion of men initially diagnosed with apparently low-risk disease on TRUSGB, subsequent TTMB revealed clinically significant PCa requiring more aggressive therapy [43]. Radical prostatectomy (RP), although the pathology gold standard reference test, could not be utilised in this study due to ethical considerations and positive selection bias.

## 5. Conclusion

A combined biopsy approach (MRI/US-TBx + TTMB) detects more significant PCa than MRI/US-TBx alone; however it will double the detection rate of insignificant PCa. MRI/US-TBx is the likely future of PCa diagnosis and in centres of excellence is a robust and valid technique. It has the potential to increase the specificity of PCa detection and reduce overdiagnosis of insignificant PCa. However, further prospective trials with longer follow-up are required, particularly for biopsy negative cases.

## Figures and Tables

**Table 1:** Baseline characteristics of MRI/US fusion targeted biopsy cohort.

	All patients (*n* = 148)	Patients with both MRI/US-TBx + TTMB (*n* = 80)
Age (median, IQR)	65 (60–70)	64 (59–69)

PSA (median, IQR)	5.0 (3.6–7.5)	5.2 (3.8–7.5)

Prostate volume (median, IQR)	44 (34–60)	42 (32–59)

Positive DRE, *n* (%)	23 (16%)	12 (15%)

Patients with previous biopsy, *n* (%)	55 (37%)	15 (19%)
Positive biopsy in the past, *n* (%)	24 (16%)	8 (10%)
Negative biopsy in the past, *n* (%)	34 (23%)	7 (9%)

ROI size in mm	7 (5–10)	7 (5–10)

Number of cores taken per location (Median, IQR)	4 (3–5)	4 (3-4)

IQR = interquartile range, *n* = number, mm = millimetres, MRI/US-TBx = MRI/transrectal ultrasound fusion targeted biopsy, and TTMB = transperineal template guided mapping biopsy.

**Table 2:** PI-RADS score and cancer detection rates (*n* = 148).

PI-RADS	Frequency	MRI-fusion biopsy	
		All PCa	Sig PCa
1/2^*∗*^	28	4 (14%)	2 (7%)
3	80	30 (38%)	17 (21%)
4	55	38 (69%)	33 (60%)
5	22	20 (91%)	18 (82%)
Total	185	92	70

PCa = prostate cancer, Sig = significant.

^*∗*^Due to clinical reasons these patients with PI-RADS 1/2 ROIs underwent targeted biopsy.

**Table 3:** Detection rate MRI/US fusion targeted biopsy by size of ROI (*n* = 148).

Lesion size	Freq.	Any PCa	Sign PCa
		MRI/US Fusion targeted biopsy	
<5 mm	22	11 (50%)	7 (32%)
5–9 mm	93	39 (42%)	26 (28%)
≥10 mm	45	28 (62%)	25 (56%)

Freq. = frequency, PCa = prostate cancer, Sign = significant, and ROI = region of interest.

**Table 4:** Detection rates for significant and insignificant prostate cancer of MRI/ultrasound fusion targeted biopsy versus transperineal template saturation mapping biopsy (*n* = 80).

	MRI/US-TBx	TTMB	*p* value (MRI versus TTMB)	CBS	*p* value (MRI versus CBS)
Any prostate cancer, *n* (%)	40 (50%)	48 (60%)	0.13	55 (69%)	<0.01
Significant prostate cancer, *n* (%)	32 (40%)	28 (35%)	0.48	39 (49%)	0.02
Insignificant prostate cancer, *n* (%)	8 (10%)	20 (25%)	0.01	16 (20%)	0.04

MRI/US-TBx = MRI/transrectal ultrasound fusion targeted biopsy, TTMB = transperineal template guided mapping biopsy, CBS = combined biopsy strategy (MRI/US-TBx + TTMB), and *n* = number.

**Table 5:** Gleason score breakdown for MRI/US-TBx and TTMB (*n* = 80).

Gleason score	MRI/US-TBx	TTMB	CBS	*p* value (MRI/US-TBx versus CBS)
No cancer	40 (50%)	32 (40%)	25 (31%)	
3 + 3	8 (10%)	20 (25%)	16 (20%)	
3 + 4	18 (22.5%)	24 (30%)	25 (31%)	
4 + 3	7 (9%)	3 (4%)	7 (9%)	
4 + 4	4 (5%)	1 (1%)	4 (5%)	
4 + 5	2 (2.5%)	0	2 (2.5%)	
5 + 4	1 (1%)	0	1 (1.5%)	
Total	80	80	80	

Low risk	8 (10%)	20 (25%)	16 (20%)	0.04
Int. risk	25 (31%)	27 (34%)	32 (40%)	0.02
High risk	7 (9%)	1 (1%)	7 (9%)	Not sig

CBS = combined biopsy strategy, MRI/US-TBx = MRI/transrectal ultrasound fusion targeted biopsy, and TTMB = transperineal template guided mapping biopsy.

Low risk = GS 6, intermediate risk = GS 7, and high risk = GS 8 or greater.

**Table 6:** Missed cancers (cancers detected on TTMB but missed on MRI targeted biopsy: significant upgrades only).

TTMB data			MRI/US fusion targeted biopsy data				
GS (% Gleason 4)	Location of cancer		GS	Location of ROI	MRI ROI location correlates to TTMB	ROI size (mm)	PI-RAD score
3 + 4 (15%)	L ant		0	L base post	No	7	2
3 + 4 (5%)	R mid post		0	L post apex	No	4	3
3 + 4 (10%)	L post apex		0	Midline post	No	11	3
3 + 4 (10%)	R base PL + R ant		0	R mid apex	No	5	3
3 + 4 (10%)	R base PL		3 + 3	R base peripheral zone	Partial	12	3
3 + 4 (2%)	L ant apex, R mid PL, L mid PL		3 + 3	L post apex	Partial	9	4
3 + 4 (5%)	R base PL		0	L base	No	2	5

MRI/US-TBx = MRI/transrectal ultrasound fusion targeted biopsy, TTMB = transperineal template guided mapping biopsy, ROI = region of interest, L = left, R = right, ant = anterior, post = posterior, and PL = posterolateral.

**Table 7:** Sensitivity, specificity, NPV, and PPV of PI-RADS scoring system for prostate cancer detection, derived from combined biopsy strategy (MRI/US-TBx + TTMB). For this analysis PI-RADS 1/2 is considered negative and PI-RADS 3–5 is considered positive. *N* = 80.

	All PCa	Sign PCa	Insig PCa
Sensitivity	96	98	91
Specificity	32	22	13
NPV	75	92	83
PPV	78	56	23

PCa = prostate cancer, Sign = significant, Insig = insignificant, NPV = negative predictive value, and PPV = positive predictive value.
